# Deep supervised hashing for gait retrieval

**DOI:** 10.12688/f1000research.51368.1

**Published:** 2021-10-12

**Authors:** Shohel Sayeed, Pa Pa Min, Thian Song Ong

**Affiliations:** 1 Faculty of Information Science and Technology, Multimedia University, Jalan Ayer Keroh Lama, Bukit Beruang, Melaka, 75450, Malaysia

**Keywords:** Gait Retrieval, Deep Supervised Hashing, Convolutional Neural Network, Binary codes

## Abstract

**Background:** Gait recognition is perceived as the most promising biometric approach for future decades especially because of its efficient applicability in surveillance systems. Due to recent growth in the use of gait biometrics across surveillance systems, the ability to rapidly search for the required data has become an emerging need. Therefore, we addressed the gait retrieval problem, which retrieves people with gaits similar to a query subject from a large-scale dataset.

**Methods:** This paper presents the deep gait retrieval hashing (DGRH) model to address the gait retrieval problem for large-scale datasets. Our proposed method is based on a supervised hashing method with a deep convolutional network. We use the ability of the convolutional neural network (CNN) to capture the semantic gait features for feature representation and learn the compact hash codes with the compatible hash function. Therefore, our DGRH model combines gait feature learning with binary hash codes. In addition, the learning loss is designed with a classification loss function that learns to preserve similarity and a quantization loss function that controls the quality of the hash codes

**Results:** The proposed method was evaluated against the CASIA-B, OUISIR-LP, and OUISIR-MVLP benchmark datasets and received the promising result for gait retrieval tasks.

**Conclusions: **The end-to-end deep supervised hashing model is able to learn discriminative gait features and is efficient in terms of the storage memory and speed for gait retrieval.

## Introduction

Gait recognition is perceived as the most promising biometric approach ^1- 3^ among behavioural biometric approaches, especially because of its efficient applicability in surveillance systems. However, gait recognition and identification tasks are becoming increasingly difficult due to the large-scale images and videos generated from surveillance systems. To ease the burden of real-world large dataset problems, researchers have applied person re-identification and retrieval approaches for surveillance video analysis or identify the person of interest. The re-identification approach finds the targeted person given by a query image from different cameras and a different time. ^4^ Similar to re-identification, gait retrieval is also used to retrieve people with similar gait from a large-scale dataset given by the query subject. In contrast, gait re-identification considers the one-to-one problem with the top-ranked item within the dataset and from the dataset, and the retrieval normally considers all the top items from the ranked list (from one to many). The retrieval problem is addressed in many biometric applications, such as face retrieval and other large-scale content searches, because of the efficiency in tracking and locating similar content. ^5^

To retrieve visually or semantically similar content, the traditional approach is to search for similar contents by ranking the contents from the database based on the similarity with the query features, and the nearest contents are returned. Nevertheless, this approach affects the computation time and memory of large-scale databases. To address these speed and storage issues, hashing methods have been proposed for use in different text, video, and image retrieval tasks. ^6^

This paper presents the deep gait retrieval hashing (DGRH) model to address the gait retrieval problem for large-scale datasets. Due to the recent growth in gait data across surveillance systems, the ability to rapidly search for the required data has become an emerging need. Linearly searching for real-value features may affect the computation time and memory storage, so the approximate nearest neighbour (ANN) search approach via hashing has attracted increasing attention. The goal of the hashing approach is to represent the input images as hash codes and learn the similarity of the learned binary codes. Similarity searching can be efficiently implemented when the high-dimensional data are transformed into compact binary codes with hashing functions. Our proposed method used the supervised hashing method with a deep convolutional network. The supervised hashing approach takes label information of each gait to generate binary codes. To extract the discriminant features from the input gait, we use a deep convolutional neural network instead of manually extracting the features. We use the convolutional neural network (CNN) to capture the semantic gait features for feature representation and learn the compact hash codes with the compatible hash function. Therefore, our DGRH model is a supervised hashing model that combines gait feature extraction and binary hash code learning. The pipeline for our proposed (DGRH) model includes:

• The sub-network of convolutional layers and pooling layers is used to extract the gait features
• The binary hash codes are generated from the last fully connected layer
• The classification and quantization loss are used to optimize the network and learn the hash function

### Related work

Similarity searching can be efficiently implemented with compact binary codes generated with hashing methods. The current hashing methods can be divided into unsupervised and supervised hashing. Unsupervised hashing methods use unlabelled data and only training data to learn the hash function and perform neighbourhood relation clustering in a Hamming space. For example, kernelized locality-sensitive hashing ^7^ uses the random projection of the hash function and constructs the kernel function to perform the similarity search. Liu *et al*. ^8^ proposed anchor graph hashing to build a neighbourhood graph that learns binary hash codes to map similarities in a Hamming space. Neighbourhood discriminant hashing (NDH) ^9^ utilizes the local discriminant information from the neighbourhood structure so that the data labels can be predicted in a Hamming space.

To reduce the complexity and perform efficient semantic similarity searching, supervised hashing approaches are practised. Supervised hashing takes advantage of label information, pairwise similarity information, or data point similarity. Liu *et al*. ^10^ proposed supervised hashing with kernels by using image pairs and converted them into binary code to map the data in Hamming distance. The kernel formulation minimizes the distance between similar pairs and maximizes the distance on dissimilar pairs. Shen *et al*. ^11^ also introduced supervised discrete hashing, which combines linear classification with the generation of hash codes into a model and addresses supervised hashing problems. Then, Lin *et al*. ^12^ proposed supervised hashing with a two-step model: binary code learning in the first step and hash function learning in the second step.

The above papers used manual feature learning, which has limitations on the diversity of the dataset and the performance. Therefore, current researchers utilize the advantages of deep networks, which can extract visual features from raw data with minimal pre-processing. Xia *et al*. ^13^ introduced a two-step paradigm for supervised hash learning with a convolutional neural network. They pre-processed the input images with a pairwise similarity matrix to give the approximated hash codes for training images and then used a convolutional neural network (AlexNet) for the feature learning of input images as well as for learning the hash codes. However, a CNN has limitations for improving hash code learning, and the one-stage method became the norm of later deep supervised hashing methods. Other researchers proposed deep supervised hashing techniques for different kinds of inputs, such as single data, paired data, and triplet pairs of data. Lai *et al*. ^14^ proposed a deep hashing architecture with triplet pair images as the input and convolutional layers to extract effective image features. They used the divide and encode module to extract the image features into branches that correspond to each hash code, and then the triplet ranking loss function was used to perceive the similarities. Thereafter, Zhang *et al*. ^15^ were inspired to use this approach for their person re-identification problem because the optimization of triplet ranking is able to capture the variation differences between the intra-class and inter-class rankings. The supervised hashing of semantic similarity based on data pairs has also received attention because of improvements in the quality of hash coding. The deep hashing network (DNH) proposed by ^16^ uses paired data for image representation and a convolutional neural network for feature extraction. The last layer of the deep network, a fully connected layer, is used to generate binary hash codes. To preserve the similarity between the pairs of images, the pairwise cross-entropy loss is adopted, and the pairwise quantization loss is adopted to control the quality of the hash code. The improved version of pairwise deep hashing is presented in DCH (deep Cauchy hashing), which uses the Cauchy distribution to design the pairwise cross-entropy loss. ^17^ The Cauchy cross-entropy loss is adapted from the Bayesian framework and well designed for the Hamming space retrieval.

To address the gait retrieval problem, Zhou *et al*. ^5^ presented the kernel-based semantic hashing method and used the Gaussian kernel function to map the gait data into the hashing function. The learned hashing function is later optimized by the triplet ranking loss, and the binary codes of gait data are stored in a database. To retrieve the given query data, the semantic ranking list is obtained based on the Hamming distance between the query data and the gait database. Rauf *et al*. ^18^ also proposed deep supervised hashing for gait retrieval using triplet pair gait data. They designed the hash model with a three-channel convolutional neural network sharing the same parameter. The hash layer is added after fully connected layers to generate the hash code. The triplet ranking loss is used for optimization, and the associated ranking list is based on labels. Their method outperformed other traditional methods because of the robustness of the CNN in visual feature learning.

### Proposed method

*Gait representation*

**Figure 1.**
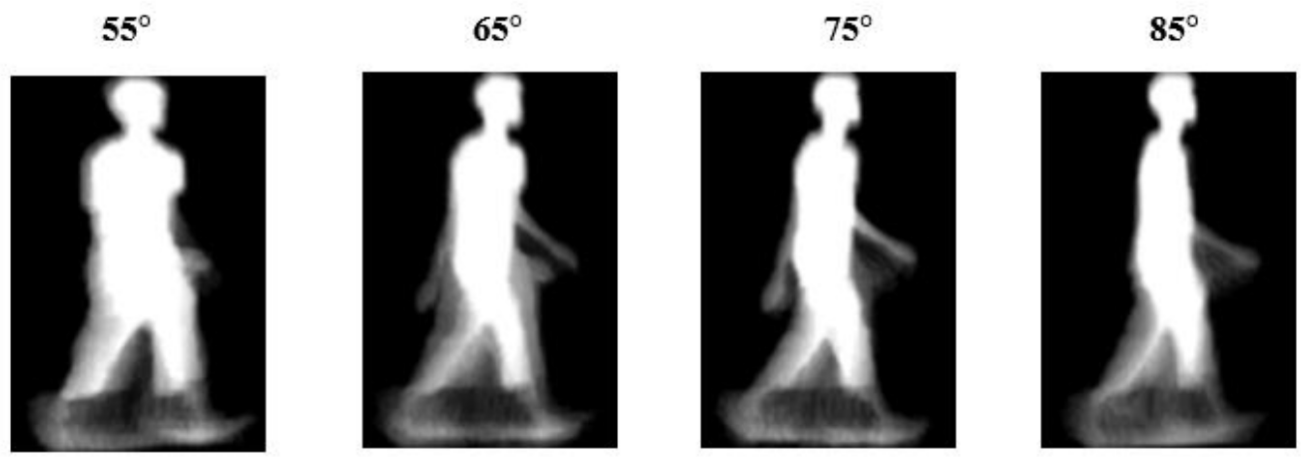
Sample gait energy image from the OUISIR-Large Population dataset.

As shown in Figure 1, we use the gait energy image (GEI). The gait energy image is a spatiotemporal gait representation that represents gait features in a single image. GEIs convert a sequence of gait silhouettes into a two-dimensional image that preserves the human motion. GEIs were first introduced by ^19^ to reduce the burden of limited gait training templates. Since the GEI can capture both temporal and spatial information, it has become the most popular gait representation. In addition, GEIs also include information on both the silhouette shape and the dynamic walking motion. GEIs can be obtained by extracting the silhouette of the human and averaging the sequence of silhouettes. The details of computing the gait energy image can be found in. ^19^

*Proposed deep gait retrieval hashing (DGRH) model*

The idea of similarity preservation in a hashing method is that similar codes are generated from semantically similar data. Therefore, mathematically, the goal of the hash function is to learn the image space X to the mapping with $H\to :X\;{\left\{-1,1\right\}}^{K}$ into the Hamming space. Here, K is the k-bit binary hash code generated from the input image X .

Suppose *N* training GEI images are given, denoted by ${\left\{x_{i}\right\}}_{i=i}^{N}$ , and each $x_{i}$ is represented by the dimension feature vector D so that $x_{i}$ $\in {\mathbb{R}}^{D}$ . Therefore, X = [ $x_{1},x_{2},x_{3},\ldots ,x_{N}]$ $\in {\mathbb{R}}^{D\times N}$ . The label matrix Y in *N* training GEIs is denoted as Y=[ $y_{1},y_{2},y_{3},\ldots ,y_{N}]$ $\in {\mathbb{R}}^{C\times N}$ , and the number of classes is denoted as *C.* If the i th GEI belongs to the j th class, $y_{\mathrm{ij}}$ = 1, and if not, $y_{\mathrm{ij}}$ = 0. Therefore, our proposed hashing method will learn the hash codes $h_{i}\in {\left\{-1,1\right\}}^{K}$ from each input GEI, where *K* is the length of the binary codes.

**Figure 2.**
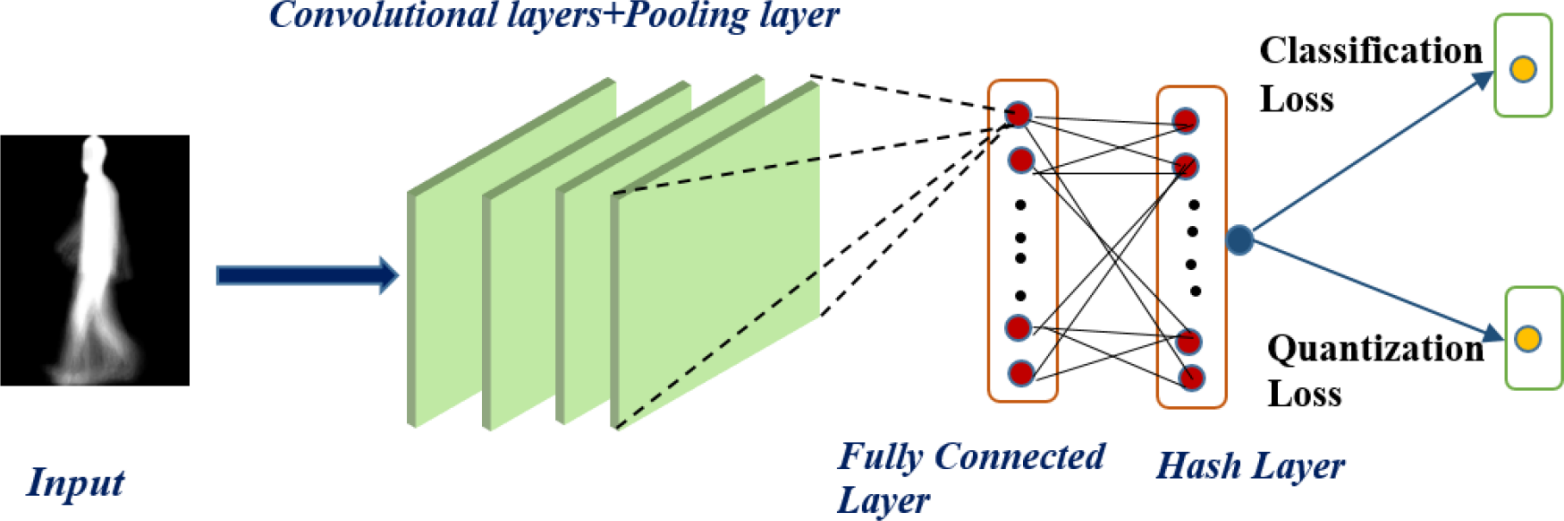
The architecture for the proposed deep gait retrieval hashing (DGRH) model.

As shown in Figure 2, our proposed model takes the gait energy image (GEI) as the input gait data and extracts the features using the deep convolutional neural network (CNN) presented by. ^20^ The architecture of the CNN is composed of convolutional and pooling layers, a fully connected layer, and a hashing layer; the last layer of the CNN network generates the binary hash code. There are four convolutional layers with different numbers of filters for each layer (16, 32, 64, 128), and the filter size for all layers is 3*3. After each convolutional layer, there is a max-pooling layer with a stride of 2. The first fully connected layer also takes the number of hidden neurons (1024) as the parameter, and the last fully connected layer serves as the fully connected hash (FCH) layer for hash function learning. The detailed architecture of the CNN proposed by ^20^ is shown in Table 1. We used the leaky rectified linear unit (LeakyReLU) activation function for all the layers except the hash layer. To learn the hash function, we used a hyperbolic tangent (tanh) activation function to compress the output of the last fully connected layers to the range of [−1,1]. The tanh function is defined as:

$$\tanh\left(z\right)=\frac{e^{z}-e^{-z}}{e^{z}+e^{-z}}$$ (1)

**Table 1.** The convolutional neural network (CNN) architecture for the proposed model.

	Network layer	Filter size	No of filters	Stride	Size of output volume
1. 2. 3. 4. 5. 6. 7. 8. 9. 10.	**Input image** **Conv1** **Pool1 (Max)** **Conv2** **Pool2 (Max)** **Conv3** **Pool3 (Max)** **Conv4** **Pool4 (Max)** **Fully connected layer 1** **Hashing layer** **(tanh)**	3 × 3 2 × 2 3 × 3 2 × 2 3 × 3 2 × 2 3 × 3 2 × 2	16 32 64 124	1 2 1 2 1 2 1 2	240× 240 × 1 238 × 238 × 16 119 × 119 × 16 117× 117 × 32 58× 58× 32 56× 56 × 64 28 × 28 × 64 26× 26 × 124 13× 13 × 124 1 ×1 × 1048 1 × 1 × K

The tanh activation function maps the data into the range of [−1,1], and the number of neurons ( *K*) for the fully connected layer is the desired length of binary hash codes (such as 16 bits, 32 bits, 64 bits, and so on). The learned hash function from the deep convolutional network is $h_{i}\in {\left\{-1,1\right\}}^{K\times N}$ with *N* GEI images. To calculate the binary hash code ( $b_{i})$ from the output of the hash layer, we utilize the *sign*(.) function; $b_{i}=\mathrm{sign}\left(h_{i}\right)$ . Here, the *sign*(.) function on a vector and a matrix is expressed as

$$\mathrm{sign}\left(x\right)=\left\{\begin{array}{l} 1,\;x\geq 0\\ 0,\;\text{otherwise} \end{array}\right.$$ (2)

*Supervised loss function and optimization*

We introduced the loss function to learn the ability of the hash codes. The designed loss functions measure the similarity-preserving ability of the hash codes. Since the well-learned hash codes have a solid classification ability, we expected that the supervised hash function can generate similar hash codes for the gait inputs with the same label. To preserve the similarity of the hash codes, we used the softmax function, which is a multinomial logistic regression function and suitable for predicting the class labels. The formulation for the softmax function is

$$P\left(Y=k|X=x_{i}\right)=\frac{e^{s_{k}}}{\sum_{j}e^{s_{j}}}\;\text{where}\;s=f\left(x_{i};W\right)$$

Here, we denote $W_{h}$ as the linear weight vectors that connect the output from the last hash layers, so $W_{h}\in {\mathbb{R}}^{k\times N}$ . The softmax linear function for the learned representation $h_{i}$ can be rewritten as

$$P\left(y\in C_{n}|h_{i};W_{h}\right)=\frac{e^{w_{n\;}^{T\;}h_{i}}}{\sum_{j=1}^{C}e^{w_{j\;}^{T\;}h_{i}}}\;$$ (3)

To minimize the loss across the training sample to further reduce the classification error, we used the cross-entropy loss function. The function of the cross-entropy loss measures the dissimilarity of the predicted label distribution with the true label probability distribution. Therefore, ${\hat{Y}}_{\mathrm{ij}}$ is the vector of the predicted label that sample *i* belongs to class *j.* The ground truth vector $Y_{\mathrm{ij}}$ is 1 if *i* belongs to class *j* and 0 otherwise. Therefore, the cross-entropy loss for ${\hat{Y}}_{\mathrm{ij}}$ and $Y_{\mathrm{ij}}$ is

$$\begin{array}{ll} L\left(Y_{ij},{\hat{Y}}_{ij}\right) & =-\frac{1}{N}\sum_{i=1}^{N}\sum_{j=1}^{C}Y_{ij}\log{\hat{Y}}_{ij}\\ & =-\frac{1}{N}\sum_{i=1}^{N}\sum_{j=1}^{C}Y_{ij}\log\frac{e^{w_{J}^{T}h_{1}}}{\sum_{k=1}^{C}e^{w_{k}^{T}h_{1}}} \end{array}$$ (4)

To reduce overfitting and the variance of the network, we introduced $L_{2}$ regularization terms to generalize the training of the deep network. Here, the regularization term is

$$R_{w}=\left(\sum_{l=1}^{L}{\left\|W^{l}\right\|}_{F}^{2}\right)\frac{\lambda }{2m}$$ (5)

where λ is the regularization parameter and ${\left\|.\right\|}_{F}^{2}$ indicates the Forbenious norm defined as ${\left\|W^{l}\right\|}_{F}^{2}=\sum_{i}\sum_{i}{\left(W_{\mathrm{ij}}^{L}\right)}^{2}=W^{T}W\;$ . Therefore, the final classification loss for our proposed model is as follows:

$$L_{C}=\;-\frac{1}{N}\sum_{i=1}^{N}\sum_{j=1}^{C}Y_{\mathrm{ij}}\log\frac{e^{w_{j\;}^{T\;}h_{i}}}{\sum_{k=1}^{C}e^{w_{k\;}^{T\;}h_{i}}}+\frac{\lambda }{2m}\left(\sum_{l=1}^{L}{\left\|W_{h}\right\|}_{F}^{2}\right)$$ (6)

*Quantization loss*

The real-value features ( $h_{i}$ ) from the hash layer need to be converted into binary codes to perform the retrieval in the Hamming space. To control the learned hash codes’ quality, we introduced the quantization loss. Discrete optimization of the classification loss function $L_{C}$ is very challenging because of the binary constraints $h_{i}\in {\left\{-1,1\right\}}^{K\times N}$ . To reduce the quantization errors, existing hashing methods apply discrete optimization to the similarity-preserving loss (classification loss) and continuous optimization to the quantization loss. The quantization error ( $Q={\left\|h_{i}-\mathrm{sgn}\left(h_{i}\right)\right\|}_{2}$ ) is optimized by applying continuous relaxation to the binary constraint. The optimization of the quantization error is very difficult due to the computation intensity and lack of compatibility with the training of the deep network with back-propagation because of the non-differentiable sgn function. Therefore, we employed the quantization loss function proposed by, ^21^ which is suitable to control the quantization error. The quantization loss is defined as follows:

$$L_{Q}={\left\|\left|h_{i}\right|-1\right\|}_{1}\;$$ (7)

$\left|.\right|$ is an elementwise vector, and we applied the smooth surrogate function to the $L_{1}$ norm.

${\left\|\left(.\right)\right\|}_{1}\approx \log\cosh\left(.\right)$ to ease the differentiation during back-propagation. The optimized quantization loss can be derived as

$$L_{Q}=\sum_{i=1}^{N}\log\cosh\left(\left|h_{i}\right|-1\right)$$ (8)

Therefore, by taking equations (7) and (9), we achieve the DGRH optimization loss:

$$\min_{W_{h}}L=L_{C}+\beta L_{Q}$$

$$\min_{W_{h}}L=-\frac{1}{N}\sum_{i=1}^{N}\sum_{j=1}^{C}Y_{\mathrm{ij}}\log\frac{e^{w_{j\;}^{T\;}h_{i}}}{\sum_{k=1}^{C}e^{w_{k\;}^{T\;}h_{i}}}+\frac{\lambda }{2m}\left(\sum_{l=1}^{L}{\left\|W_{h}\right\|}_{F}^{2}\right)+\beta \sum_{i=1}^{N}\log\cosh\left(\left|h_{i}\right|-1\right)$$ (9)

By optimizing the classification loss ( $L_{C}$ ), we can learn similarity-preserving quality hash codes and control the learned hash codes with the joint optimization function quantization loss ( $L_{Q})$ . The k-bit binary codes are achieved using the sgn function, and the final DGRH optimization loss function is able to reduce the quantization error and increase the retrieval performance. Our proposed DGRH model is trained using adaptive moment estimation (Adam) via back-propagation. In the testing stage, the new query image is converted into hash codes by the trained network. The k-bit binary codes can be obtained using the sgn function. Once we obtain the k-bit binary codes from the gallery and query sets, we can compute the Hamming distance between them to obtain the result.

## Methods

### Implementation of proposed method

The proposed model is developed using the Python programming language. For the data-pre-processing, the Pandas library was used to create and analyse the training and testing gait dataset, and Pickle library was used to import and load the data. The proposed model uses the deep learning approach i.e. the convolutional neural network. To build the convolutional neural network, the deep learning framework known as Keras library with the TensorFlow backend was used. The Keras library allows to construct the neural network easily, and can perform the training and testing of the proposed model. The other important libraries that were used in the development of the model are Numpy and Math libraries for array data manipulation and mathematical formula construction. Finally, the Matpoltlib and Seaborn libraries were used for the data visualization of the performance. The source code used for the analysis can be found at Zenodo. ^25^

### Datasets and experimental setting

We evaluated our proposed deep gait retrieval with public benchmark datasets (CASIA-B, OUISIR-LP, and OUISIR-MVLP). Since we are dealing with the retrieval problem, we considered both short-term and long-term retrieval. For short-term retrieval, we divided the datasets into the same conditions (being in view, wearing clothes, carrying a bag) since gait is captured from the camera in a short amount of time. In real-world gait applications, people are captured at different times and views from different cameras. Therefore, we considered the prepared dataset for both same and mixed conditions to further evaluate our proposed gait retrieval framework.

*CASIA-B dataset*

The CASIA-B gait dataset contains 124 subjects with 11 views and three walking conditions (normal walking, wearing a coat and carrying a bag). ^22^ We evaluated only the same view (90 ° ) with the same walking condition for this setting. For the normal walking condition, four walking sequences (nm1- nm4) are used for the training set, and the remaining sequences (nm5 and nm6) are used for the testing set. There are only two walking sequences for both wearing a coat (cl1, cl2) and carrying the bag (bg1, bg). Therefore, we prepared cl1 as the training set and cl2 as the testing set. Likewise, bg1 was used for training and bg2 was used for testing in the condition of carrying a bag. To evaluate the long-term retrieval problem, we mixed three conditions (NM, CL, BG) and prepared the datasets. The training sets (nm1, nm2, nm3, nm4, cl1, bg1) are included, and for the testing set, we used nm5, nm6, cl2, and bg2.

*OUISIR Large Population (LP) dataset*

To evaluate the proposed framework, we used a subset of the OULP datasets with 1912 subjects and 4 different views ( 55°,65°,75°,and85° ) following the protocol in. ^23^ The training and testing dataset is divided equally with 956 subjects each. Therefore, each subject also has eight GEIs with 4 views and 2 sequences. For the evaluation under the same condition, we prepared the datasets with only the same views ( 55°−55°,65°−65°,75°−75°,and85°−85° ). Under mixed conditions, each subject with 4 different views is combined for evaluation. Therefore, each subject has eight GEIs (2 walking sequences * 4 views) to perform long-term gait retrieval.

*OUISIR-Multiview Large Population (MVLP) dataset*

OUISIR-MVLP includes 10307 subjects; 5114 subjects are males, and 5193 are females. For the experiments in the OU-MVLP dataset, we followed the protocol setting of ^24^ which divided the dataset into nearly equal groups with 5153 subjects for the training set and 5154 subjects for the testing set. As we pre-processed the gait sequences into gait energy images with normalized dimensions ( 128×88) , we obtained 28 GEIs with 14 different views and 2 walking sequences for each. To evaluate our proposed method, we used only four views (0 ° , 30 ° , 60 ° , and 90 ° ) for both the same-condition and mixed-condition settings since the GEIs with 180 ° view differences are flipped versions of the images and are considered same-view pairs based on the perspective of the projection. Only the same view was used to perform the short-term gait retrieval. Under mixed conditions, we created datasets with all four views for each subject. Therefore, the number of subjects was still the same, and each subject had eight sequences (2 walking sequences * 4 views).

### Evaluation criteria

We adopted the Hamming space retrieval approach to evaluate the performance of our proposed model. In hashing, similarity-preserving hash codes are represented instead of data points to increase the speed of retrieval and reduce the storage space. The common methods for searching hash-based codes are the Hash lookup table and the Hamming ranking. The Hamming ranking uses the Hamming distance between the query image and the images from the database. The Hamming distance is computed using a bitwise operation, and the ranked list is generated according to the distance. The returned ranked list is in ascending order with the nearest neighbour in the database with the query. For the hash lookup table approach, lookup tables are constructed with the data points in the database within the Hamming radius ( r ) of the query. Hash lookup, also known as bucket searching, tries to retrieve all *r*-neighbours for each query.

The k-bit hash code lengths for the proposed model are 16 bits, 32 bits, 48 bits and 64 bits. The retrieval results are evaluated in three different matrices: precision curves with respect to the Hamming radius, precision curves based on different returned images and the mean average precision (MAP). The precision of *r* (P@r) is calculated, and the accuracy of the returned images based on the Hamming distance of the query and database images is less than or equal to r ( ≤r). Here, we set r=2 for the hash lookup and constructed the precision curves within the Hamming radius ( P@r=2) with respect to different bit lengths. In Hamming ranking, the precision is calculated by the given top returned image. Therefore, we analysed the precision curves with different numbers of top returned images (P@N).

The MAP (mean average precision) is the most important metric to evaluate the hashing algorithms. The ranked list achieved by the Hamming distance between the database and each query is evaluated against the given top returned images. First, we compute the average precision for each query with

$$\mathrm{AP}@N=\frac{\sum_{n=1}^{N}P\left(n\right)\delta \left(n\right)}{\sum_{n^{\prime }=1}^{N}\delta \left(n^{\prime }\right)}\;$$ (10)

where *N* represents the top returned images in Hamming ranking and $P\left(n\right)$ is the precision of the top-N retrieved results. Then, $\;\delta \left(n\right)$ is 1 if the n-th retrieved result is in the list and $\delta \left(n\right)$ = 0 otherwise. Then, we can calculate the mean AP for all the testing queries to obtain the mean average precision (MAP). The larger the number of MAPs is, the better the quality of retrieval performance.

## Results and discussion

**Figure 3.**
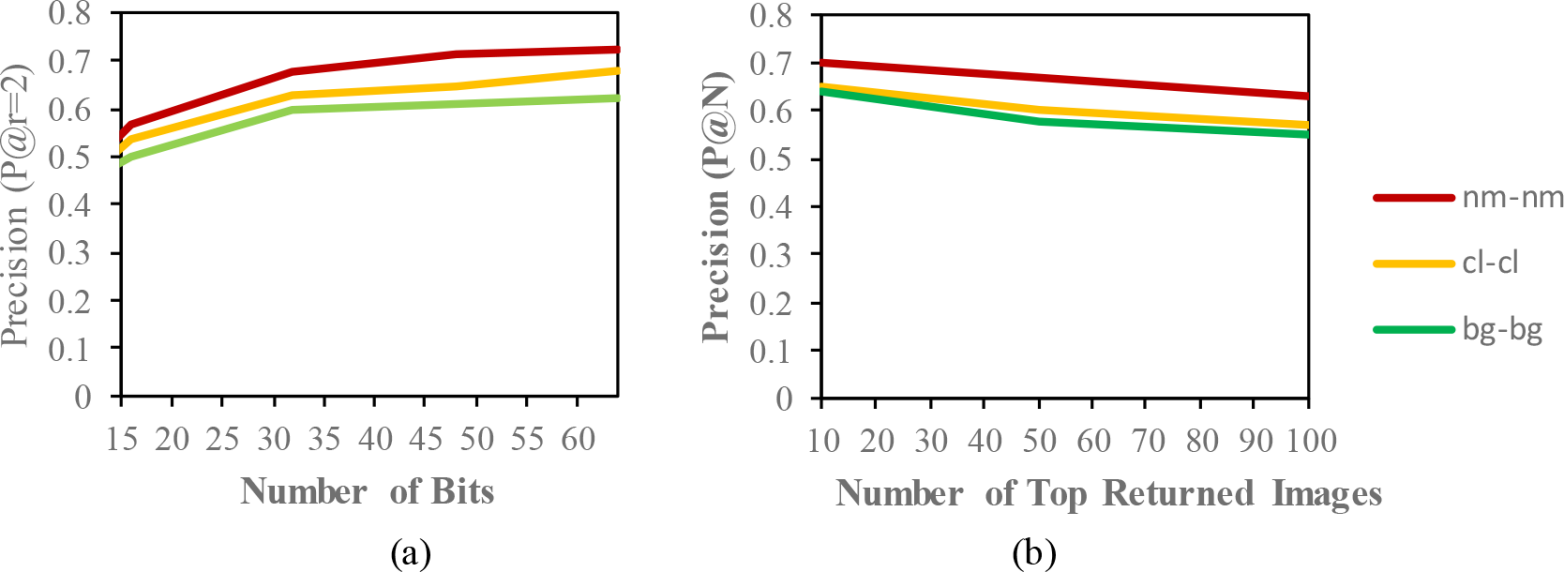
Comparison of the (a) precision curves at a Hamming radius of 2 (P@r = 2) with different bit lengths and the (b) precision curves of the top-N returned images on the CASIA-B dataset.

**Figure 4.**
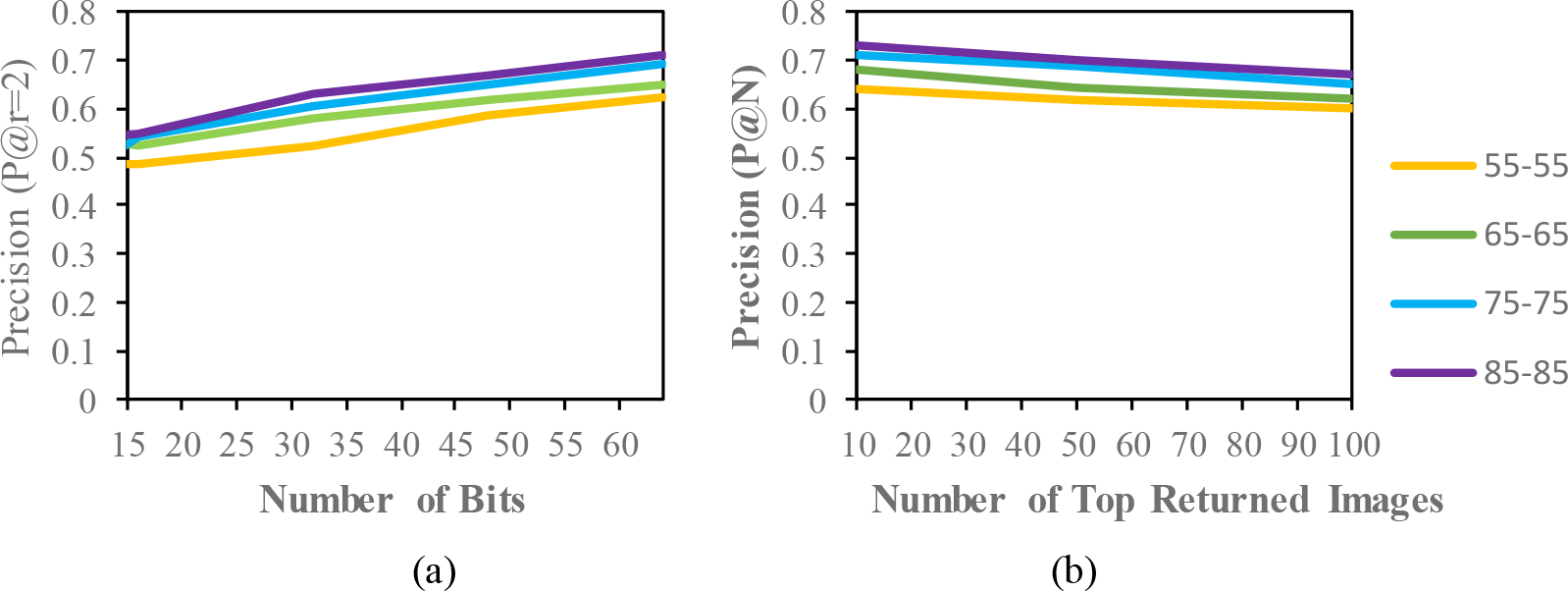
Comparison of the (a) precision curves at a Hamming radius of 2 (P@r = 2) with different bit lengths and the (b) precision curves of the top-N returned images on the OUISIR-LP dataset.

**Figure 5.**
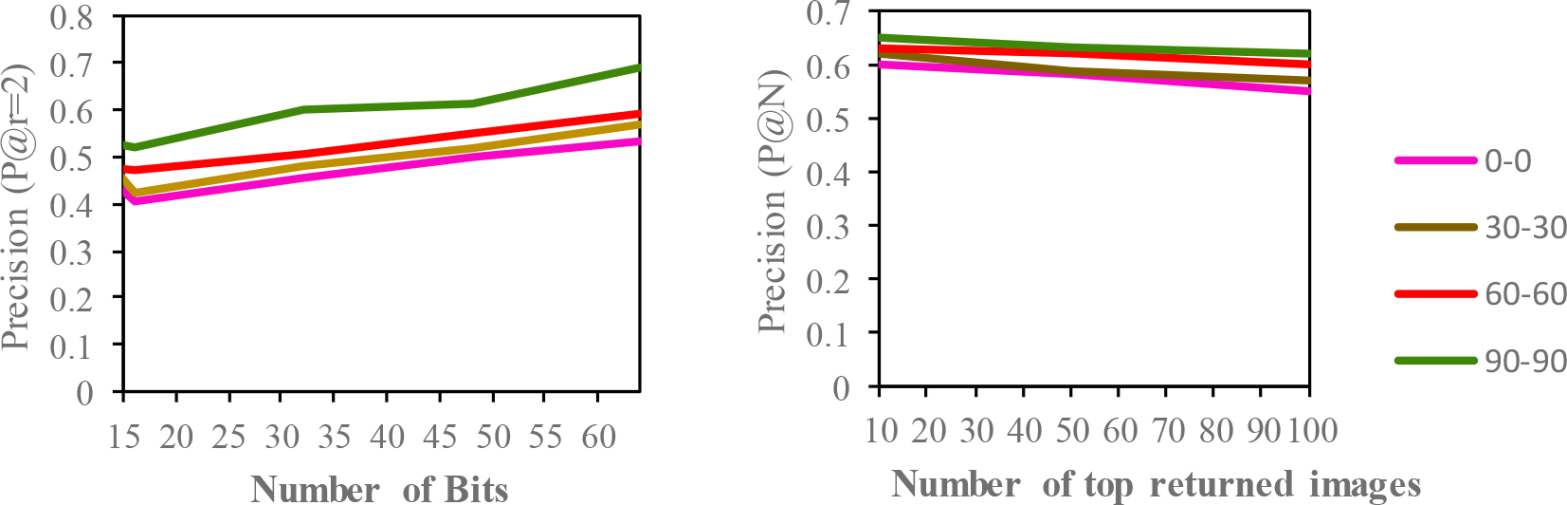
Comparison of the (a) precision curves at a Hamming radius of 2 (P@r = 2) with different bit lengths and the (b) precision curves of the top-N returned images on the OUISIR-MVLP dataset.

The mean average precision (MAP) of different datasets with the code length (16,32,48,64) for the same condition are described in Table 2, Table 3 and Table 4. In these tables, the MAP is calculated from the top-100 returned images from the given queries. The precision curves within the Hamming radius ( P@r=2) are also illustrated for different code lengths in Figures 3 to 5. Additionally, based on the top-N returned images, precision curves are also shown for further evaluation of the proposed model.

**Table 2.** MAP of the CASIA-B dataset in the same-condition setting.

	Mean average precision (MAP)			
Dataset	16 bits	32 bits	48 bits	64 bits
**nm-nm**	0.62	0.67	0.78	0.84
**cl-cl**	0.57	0.63	0.76	0.81
**bg-bg**	0.56	0.59	0.68	0.78

**Table 3.** MAP for the OUISIR-LP dataset in the same-condition setting.

	Mean average precision (MAP)			
Dataset	16 bits	32 bits	48 bits	64 bits
**CASIA-B**	0.69	0.68	0.81	0.87
**OUISIR-LP**	0.81	0.84	0.97	0.98
**OUISIR-MVLP**	0.47	0.53	0.56	0.65

**Table 4.** MAP for the OUISIR- MVLP dataset in the same-condition setting.

	Mean average precision (MAP)			
Dataset	16 bits	32 bits	48 bits	64 bits
**0-0**	0.32	0.39	0.43	0.48
**30-30**	0.48	0.54	0.56	0.52
**60-60**	0.47	0.51	0.57	0.59
**90-90**	0.51	0.49	0.54	0.56

According to the results, the CASIA-B dataset has the lowest MAP values for the carrying condition (bg-bg) since the motion features of the gait are affected by human walking motion. In addition, normal walking (nm-nm) achieves the highest MAP because of its uncorrupted gait features, and the clothing condition (cl-cl) achieves the second-best result. The MAP for the OUISIR-LP dataset is quite similar for all of the views since the motion features of the different views are observable in the side view. The highest MAP values are achieved at (85°) and a 64-bit code length. Compared to the other datasets, the MAP of the OUISIR-MVLP datasets is the lowest overall for the training and testing pairs since this dataset has the largest number of subjects. The highest MAP is in the lateral view pair (90°–90°), and the lowest is in the frontal view pair (0 °−0° ). For the bitwise comparison, the hashing performance is better for 48 bits and 64 bits. As the bit length increases, the MAP also increases for the proposed model. Therefore, the precision curves @ top-N are constructed based on the precision values at 64 bits with different returned images (10, 50, 100), as shown in Figure 5.

**Figure 6.**
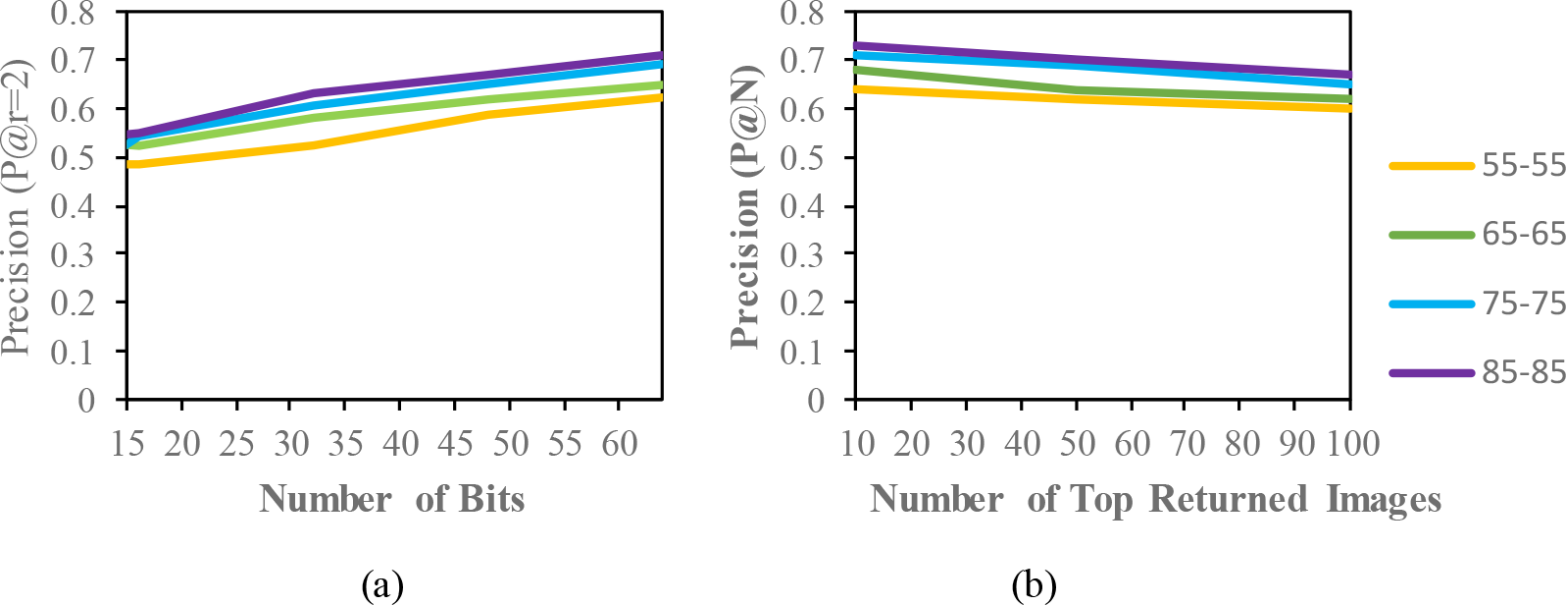
Comparison of the (a) precision curves at a Hamming radius of 2 (P@r = 2) with different bit lengths and the (b) precision curves of the top-N returned images on the different datasets.

The evaluation result for the mixed condition is shown in Table 5. The purpose of the mixed condition is to address long-term gait retrieval. The CASIA-B dataset explores different walking conditions, such as carrying bags and wearing conditions, in the same view. According to the results, the MAP of the mixed condition in the CASIA-B dataset is able to achieve a higher performance than the same condition. Hamming distance is able to overcome the covariate factors in the retrieval task. To analyse the retrieval performance in view changes, the OUISIR-LP dataset and OUISIR-MVLP datasets are subjected to experiments with four different views. The angular difference for the OUISIR-LP is 10°, and the largest difference is only 30°. Therefore, the MAP for the OUISIR dataset is the highest among other datasets, and the results of the precision curves for a Hamming radius of 2 and top-returned images are also desirable. The MAP for the OUISIR-MVLP dataset is the lowest among the datasets, which is probably due to the large angular difference in the view changes. The gait is captured from both frontal and lateral views ranging from 0° to 90°. The motion and gait features might not be observed well in the frontal view compared to the lateral view. However, the retrieval performances are quite desirable given the large population dataset with larger view changes. In terms of the bit comparison, the 64-bit scheme also achieves better precision results than the other bit lengths. Therefore, the precision curves for the top-returned images are illustrated based on the precision of 64 bits, and the results are shown in Figure 6. According to the result, the highest precision value based on the different number of bits is from the OUISIR-LP dataset with 4 different views and small angular differences. The CASIA-B dataset is also able to achieve the desired result, and the lowest value is for the OUISIR-MVLP dataset. In Figure 6, we compare the precision values based on the number of top-returned images in 64 bits. The OUISIR-LP dataset is also able to achieve a high precision value, while the OUISIR-MVLP dataset has the lowest precision with a large number of angular views.

**Table 5.** MAP for different datasets with the mixed condition.

	Mean average precision (MAP)			
Dataset	16 bits	32 bits	48 bits	64 bits
**55-55**	0.59	0.57	0.62	0.64
**65-65**	0.64	0.63	0.68	0.72
**75-75**	0.61	0.66	0.68	0.74
**85-85**	0.69	0.76	0.74	0.78

### Comparison with other existing methods

The performance of the DGRH (deep gait retrieval hashing) method is compared against the current gait retrieval works with the same datasets. There are only two papers that address the gait retrieval problem. ^5, 18^ Therefore, the first method to be evaluated is the kernel-based semantic hashing method (KSH) proposed by Zhou *et al*. ^5^ The KSH uses the handcrafted feature learning approach with the Gaussian kernel function to generate the hashing function, and a semantic ranking list is used to retrieve the gait data. Another gait retrieval method that is compared is the deep hashing method presented by Rauf *et al*., ^18^ who used deep supervised hashing methods with triplet deep learning channels. This method takes the triplet pairs of gait data into the shared triplet channel to calculate the hash function, and the triplet ranking loss is used to retrieve the query from the ranking list.

**Figure 7.**
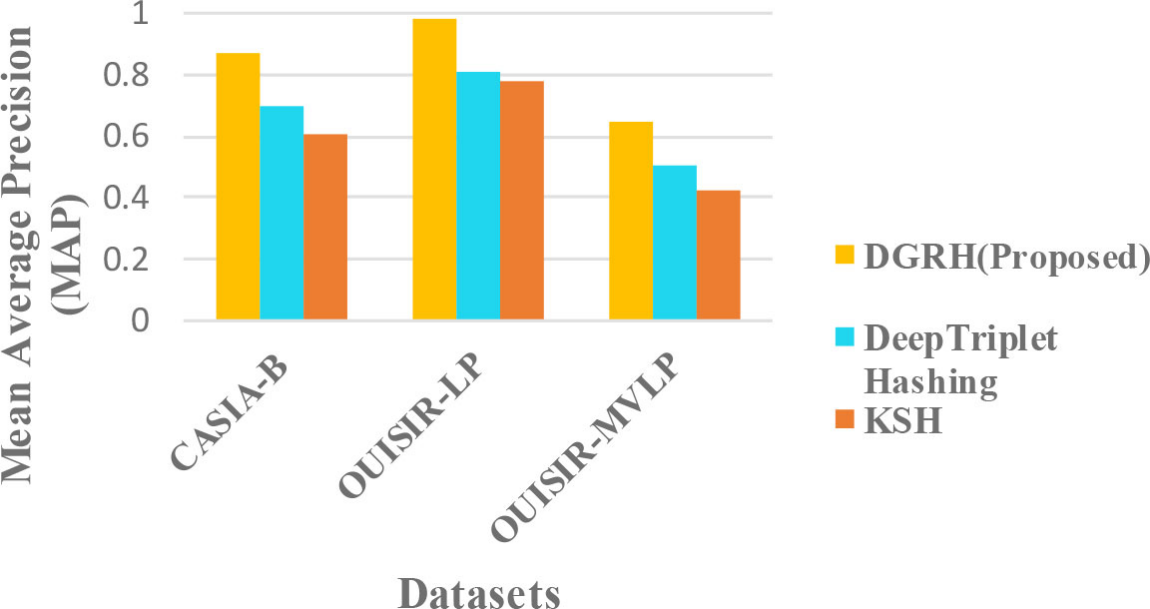
Comparison of the mean average precision MAP in different methods.

The proposed framework is compared with the existing methods in Figure 7. The mean average precision (MAP) of the top-100 returned images is used as the evaluation criterion for the retrieval performance.

The mixed condition in different datasets is analysed since gait retrieval is mostly a long-term retrieval problem. According to the results, our proposed method outperforms the other two methods. The retrieval performance of our proposed method is better because of the deep representation of the gait features, and the strength of the CNN can learn better information about the gait motion. The pairwise-based or triplet-based loss in hashing might cause a data imbalance problem because of its complex data preparation for suitable data pairs. In addition, this approach can also suffer from optimization problems. The combination of the classification loss and quantization loss in the proposed method can effectively predict gait labels and control the quality of the hash codes.

## Conclusion

This paper proposed the deep gait retrieval hashing (DGRH) model to address gait retrieval. The DGRH uses supervised deep hashing to retrieve the individual gait from the given query. The deep convolutional neural network is used to extract the gait features and generate the hash codes from the last layer of the network. The hash function is learned by optimization of the classification loss and quantization loss, and then gait retrieval is performed in the Hamming Space. The end-end-end hashing model is able to learn discriminative gait features and is efficient in terms of the storage memory and speed. The proposed method is evaluated on three different public datasets and outperforms other methods.

## Data availability

### Underlying data

CASIB-B Dataset: The dataset is provided by The Institute of Automation, Chinese Academy of Sciences (CASIA) for the research purposes. We used the dataset B from the CASIA Gait Dataset which available on http://www.cbsr.ia.ac.cn/english/Gait%20Databases.asp by signing the release agreement.

OU-ISIR LP Dataset: The OU-ISIR Gait Database, Large Population Dataset is provided by The Institute of Scientific and Industrial Research (ISIR), Osaka University (OU). We used that dataset from http://www.am.sanken.osaka-u.ac.jp/BiometricDB/GaitLP.html by signing the release agreement for research purposes.

OU-ISIR MVLP Dataset: The OU-ISIR Gait Database, Multi-View Large Population Dataset (OU-MVLP) is provided by The Institute of Scientific and Industrial Research (ISIR), Osaka University (OU). We used the dataset from http://www.am.sanken.osaka-u.ac.jp/BiometricDB/GaitMVLP.html by signing the release agreement for research purpose.

All the datasets can be obtained by signing the release agreement under research purpose.

## Software availability

Source code available from: https://github.com/papamin/Deep-Supervised-Hashing-for-Gait-Retrieval/tree/v1.0.1.

Archived source code at the time of publication: https://doi.org/10.5281/zenodo.5256521. ^25^

License: GPL 3.0.
