# Supplementary material for: Deep supervised hashing for gait retrieval
Source: F1000Res. 2022 Jun 22;10:1038. Originally published 2021 Oct 12. [Version 2] doi: 10.12688/f1000research.51368.2 (PMC9237558; doi:10.12688/f1000research.51368.2)
Supplement: Peer review history

# Peer review: Reviewer response for version 2

**DOI:** 10.5256/f1000research.134760.r141676

**Published:** 2022-06-27

**Authors:** Md. Rajibul Islam

**Affiliations:** 1 Department of Computer Science and Engineering, University of Asia Pacific, Dhaka, Bangladesh

The authors have addressed my concerns. The manuscript quality has been greatly increased with all the modifications to the manuscript. I believe it currently meets the standards expected for F1000Research articles.

Is the rationale for developing the new method (or application) clearly explained?

Yes

Is the description of the method technically sound?

Yes

Are the conclusions about the method and its performance adequately supported by the findings presented in the article?

Yes

If any results are presented, are all the source data underlying the results available to ensure full reproducibility?

Yes

Are sufficient details provided to allow replication of the method development and its use by others?

Yes

Reviewer Expertise:

Machine learning, Artificial intelligence, Data mining.

I confirm that I have read this submission and believe that I have an appropriate level of expertise to confirm that it is of an acceptable scientific standard.

# Peer review: Reviewer response for version 1

**DOI:** 10.5256/f1000research.54529.r96765

**Published:** 2021-12-15

**Authors:** Md. Rajibul Islam

**Affiliations:** 1 Department of Computer Science and Engineering, University of Asia Pacific, Dhaka, Bangladesh

This study demonstrates the deep gait retrieval hashing (DGRH) model to address the gait retrieval problem using public benchmark datasets (CASIA-B, OUISIR-LP, and OUISIR-MVLP). This method is based on a supervised hashing method with a deep convolutional network. I think this paper proposes an interesting scheme of gait retrieval. The analysis process and results presented in this paper are technically sound. I do have a few comments for the authors. I would recommend it being indexed if these were addressed.

1. Supervised hashing methods with a well-designed deep convolutional neural network have been used by various researchers in the literature. However, the authors have compared their results with only two papers that demonstrated hashing method to address the gait retrieval problem. What about the other techniques that had been used to solve the gait retrieval problem? Does the proposed method outperform those techniques too?
2. “Comparison with other existing methods” section requires improvement. The comparison statements in this section should be supported by analytical outcomes. For instance, it should be mentioned that which outcome of this article demonstrates that data imbalance and optimization problems can be solved by the proposed method and how.
3. The statement, “The hashing method is efficient in terms of the storage memory and speed” has been written at the beginning and end of the article but no results/theoretical explanation has been presented to support it.

Is the rationale for developing the new method (or application) clearly explained?

Yes

Is the description of the method technically sound?

Yes

Are the conclusions about the method and its performance adequately supported by the findings presented in the article?

Yes

If any results are presented, are all the source data underlying the results available to ensure full reproducibility?

Yes

Are sufficient details provided to allow replication of the method development and its use by others?

Yes

Reviewer Expertise:

Machine learning, Artificial intelligence, Data mining.

I confirm that I have read this submission and believe that I have an appropriate level of expertise to confirm that it is of an acceptable scientific standard, however I have significant reservations, as outlined above.

# Peer review: Reviewer response for version 1

**DOI:** 10.5256/f1000research.54529.r96767

**Published:** 2021-10-28

**Authors:** D. I. George Amalarethinam

**Affiliations:** 1 Jamal Mohamed College, Tiruchirappalli, Tamil Nadu, India

- The salient features of gait are analysed using three different benchmark dataset in connection with storage memory and speed.
- The data Tables 3 , 4, and 5 need to be spelled out so as to provide more clarity towards further proceedings by others.
- Though the paper is technically sound, the limitations or negative aspects of the proposed methodology need to be included.
- The recent year (2020) references may be provided towards strengthening of the paper.

Is the rationale for developing the new method (or application) clearly explained?

Yes

Is the description of the method technically sound?

Yes

Are the conclusions about the method and its performance adequately supported by the findings presented in the article?

Partly

If any results are presented, are all the source data underlying the results available to ensure full reproducibility?

Yes

Are sufficient details provided to allow replication of the method development and its use by others?

Partly

Reviewer Expertise:

NA

I confirm that I have read this submission and believe that I have an appropriate level of expertise to confirm that it is of an acceptable scientific standard.
